# Memoir of Dr. S. A. Cartwright, of N. O., La., Formerly of Natchez, Miss.

**Published:** 1873-12

**Authors:** J. W. M. Shattuck

**Affiliations:** Mississippi


### MEMOIR OF DR. S. A. CARTWRIGHT, OF N. O., LA., FORMERLY OF NATCHEZ, MISS.

BY J. W. M. SHATTUCK, OF MISSISSIPPI.

**Read before the Columbus and Lowndes County Medical Society.**

Of this distinguished physician and scientific investigator, there has been no complete or extended biography published.

In the N. O. Medical and Surgical Journal for Nov., 1866, (Vol. XIX, No. 3,) we find a brief memoir, from which we learn that Dr. Samuel Adolphus Cartwright was a native of Fairfax county, Va.; a son of Rev. Jno. S. Cartwright. He was born in 1793; studied medicine under the celebrated Dr. Benjamin Rush, of Philadelphia, and received his degree from the University of Pennsylvania. Dr. Cartwright began the practice of medicine in Huntsville, Ala., and removed to Natchez, Miss., in 1823, making that city his home and field of labor for a full quarter of a century.

In 1825, he married a daughter of Dr. Woodson Wren, of Natchez, and soon became an eminent and leading physician and medical writer; his articles were published and copied by most of the medical journals of that period, and attracted much attention both in this country and Europe.

Dr. Cartwright sought new and unfrequented fields of research; he was not satisfied to follow at all times in the old, beaten tracks of the profession, but boldly laid out new routes for himself to pursue, advancing new theories, supporting and defending them with science and experience; his reasoning was clear, forcible and logical.

"His writings," says one of his admirers, "are scattered through the periodical literature of the Southern States for the last forty years.

With other scientists, Dr. Cartwright claimed that the "prinum mobile" of the circulation and the chief motion power of the blood, was in the lungs and not in the heart, and he made many vivisections and successful experiments to prove the correctness of his theory.

He received several valuable medals and prizes for essays on medical topics, including the subject of yellow fever, and cholera infantum. His success in the treatment of yellow fever was unprecedented, but the full power and scope of his giant mind and untiring energy, were much more manifest in doing battle against that then (1832) new and terrible pestilence, Asiatic Cholera, which appeared in Natchez and other American cities in 1832. In contending with the deadly ravages of that fearful malady, his zeal, skill and benevolence were at once enlisted. On this point, we learn from the article alluded to that his extraordinary success in the treatment of Asiatic cholera attracted the attention of the medical profession both in this and foreign countries.

His opinion regarding its treatment was most eagerly sought for at the next visitation of that dreaded scourge. Many honors were tendered and urged upon him for his learning and abilities as a physician. He was offered a Professorship in several of the leading medical colleges of that day. These he declined, preferring to continue in the regular duties of his chosen profession, believing he could be more useful there than in any other sphere of action.

Among the many testimonials Dr. Cartwright received for his learning, benevolence and skill, was one which is not generally known, even by the profession, yet should be recorded and published in justice to his memory, and the appreciation of his merits by his friends and the public. We allude to the munificent gift bestowed by the liberality and esteem of his many friends and the authorities of the city of Natchez. This was a gold tankard, costing one thousand dollars, bearing the following inscription :

"Presented to Dr. Samuel A. Cartwright, of Natchez, Mississippi, by the planters of Adams County, as a testimonial of their friendship and grateful sense of his medical talent, especially as evidenced in his successful treatment of the cholera, which so recently and to such an alarming extent existed among them, July, 1833." On the reverse side of the vase, in bass relief, is the story of the Good Samaritan. This highly valued tribute of friendship and confidence is now properly cared for by Mrs. W. Alexander Gordon, of New Orleans, a daughter of Dr. Cartwright. To her kindness and assistance we are indebted for this record of that pleasant incident in her father's life. It was a well merited and appropriate compliment to a worthy man.

In 1836 and 1837, having acquired an ample competence, Dr. Cartwright, with his family, visited Europe. He was cordially received by the medical faculty of the principal cities. His theories had gone before him, and been adopted in practice. His writings have always attracted much attention from their peculiar style and originality.

During the financial crash of 1837, his fortune, like thousands of others, was lost in the general ruin; the earnings of a lifetime were gone in a day. Yet he was not discouraged. He returned to his impoverished and desolate home, and at the age of forty-four, like a hero began anew his professional labors, exhibiting still another trait of a noble, manly character.

In 1848, Dr. Cartwright removed to New Orleans, where he rapidly advanced to the front rank of the medical profession, as an eminent physician and scientist. He continued in active practice until his health began to fail in 1862. He then visited his old friends in Mississippi. While there he was requested by President Davis to give his advice and assistance towards improving the sanitary condition of the troops in and around Port Hudson and Vicksburg, and he was in the discharge of this duty when death called him hence.

"His last days, as all his life had been, were passed in doing good." In the death of Dr. S. A. Cartwright his immediate relatives lost a kind and loving protector; his patients, a true friend and benefactor; his professional brethren, one of their most learned and skillful members and associates, and medical literature lost one of its ablest contributors.

No higher encomium or more desirable and enduring testimonial can be offered than Dr. S. A. Cartwright has received. Of him it has been said: "Full of charity and good will towards all men, he fulfilled faithfully his station in all the relations of life."—*Trans. Am. Med. Association.*

---

THE DELIVERY OF THE PLACENTA BY SUPRA-PUBIC PRESSURE. —Judging from our own experience, and from the number of laudatory papers on this subject, Crede's method of delivering the placenta, or some slight modification of it, bids fair to take the place of every other. The plan which we adopt is as follows:

At the maximum of the first uterine contraction after birth of the child, the fundus of the womb is grasped through the abdominal wall, between the thumb in front and the fingers behind. It is then to be both forcibly squeezed and at the same time pressed downward and backward. By means of this uterine expression the placenta and membranes are usually at once detached and extruded. Sometimes, indeed, they will suddenly pop out of the vulva, just as the stone escapes when a cherry is compressed between the finger and thumb. Occasionally it will require two or more pains to effect this; but the sooner this plan is resorted to after the birth of the child, the more easy in execution will it be. Those who like ourselves, practice this method, contend that it offers many advantages over any other. The risk of communicating any puerperal disease is lessened. The expulsion of the placenta and membranes by a *vis a tergo* is more likely to be complete than by traction on the cord, which cannot be broken, as no traction is made on it. Adherent placenta is less frequently met with. The introduction of the hand into the womb is avoided, and so also, as a consequence, is the ingress of air. Finally, the tonic and energetic contraction of the womb, following this manœuvre, prevents the occurrence of hemorrhage or of unruly after-pains.—*Goodell, in Transactions Med. Society, Penn.,* June, 1873.

TINCTURE OF IODINE IN VOMITING.—Schneider, of Offenburg, administered tincture of iodine in doses of ten drops, on sugar, thrice daily, to a patient who was troubled with salivation and vomiting after intermittent fever. Although the vomiting had proved rebellious to all other treatment, it yielded to tincture of iodine.—*The Doctor.*

